# MicroRNA-18a-5p functions as an oncogene by directly targeting IRF2 in lung cancer

**DOI:** 10.1038/cddis.2017.145

**Published:** 2017-05-04

**Authors:** Chen Liang, Xing Zhang, Hui-Min Wang, Xiao-Min Liu, Xin-ju Zhang, Bo Zheng, Guang-Ren Qian, Zhong-Liang Ma

**Affiliations:** 1 Lab for Noncoding RNA & Cancer, School of Life Sciences, Shanghai University, Shanghai 200444, China; 2 School of Environmental Science and Engineering, Shanghai University, Shanghai, 200444, China

## Abstract

Lung cancer is the major form of cancer resulting in cancer-related mortality around the world. MicroRNAs are endogenous small non-coding single-stranded RNAs, which can engage in the regulation of gene expression. In this study, miR-18a-5p significantly upregulated in non-small cell lung cancer (NSCLC) tissues and NSCLC cell lines, suggesting an oncogenic function in lung cancer. Additionally, miR-18a-5p can promote carcinogenesis by directly targeting interferon regulatory factor 2 (IRF2). Further experiments indicated that IRF2 can increase cell apoptosis, inhibit cell proliferation and migration ability. Our study demonstrates that miR-18a-5p promotes autophagy in NSCLC. Collectively, these results indicate that miR-18a-5p can not only promote NSCLC by suppressing IRF2, but also will be a promising target in the near future.

Lung cancer is one of the most common malignant tumours, the new cases accounting for 13% of total cancers, and the leading cause of cancer death.^1^ The non-small cell lung cancer (NSCLC) contributes to nearly 80% of lung cancer, and its overall 5-year survival rate is low while recurrence rates remains high.^2^ For these factors, further understanding of the mechanisms involved in the tumorigenesis of NSCLC and efforts to reveal and promote potential therapeutic targets is necessary for advancement in the treatment of this prevalent disease.

MicroRNAs (miRNA) are endogenous small non-coding single-stranded RNAs, which regulate gene expression by binding to the 3'-untranslated region (3'-UTR) of mRNA.^3, 4^ Biological processes, including growth, differentiation, apoptosis, motility and malignant transformation are profoundly influenced by miRNAs.^5^ Several studies have shown ectopic expression of miR-18a-5p in cancers such as breast cancer, hepatocellular carcinoma, prostate cancer and other cancers.^6, 7, 8, 9^

The interferon regulatory factor (IRF) proteins family is the key factor of adaptive immunity, and modulated cellular responses that is involved in tumorigenesis.^10, 11^ IRF2 is a member of the IRF family, which has the ability to exert anti-oncogenic activities. As described above, elevated IRF2 expression levels leaded to reduced PD-L1 expression and associated with a decreased proliferative potential.^12^ Consistently, overexpression of IRF2 led to a dramatic cell death response by apoptosis in hepatocellular carcinoma.^13^

In this study, we investigated the potential mechanisms of miR-18a-5p in NSCLC. Simultaneously, our experimental data revealed that miR-18a-5p significantly promoted NSCLC tumour growth and migration through targeting IRF2. Our results provided a new potential direction for NSCLC diagnosis and therapeutic intervention.

## Results

### MiR-18a-5p is upregulated in NSCLC

**Figure 1:**
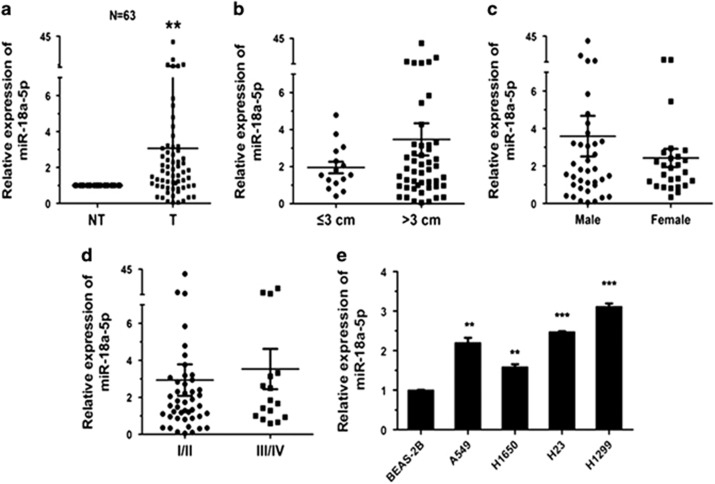
MiR-18a-5p is upregulated in NSCLC tissues and cell lines (**a**) The relative expression level obtained from the qRT-PCR results of miR-18a-5p in tumour tissues (T) and corresponding non-tumour tissues (NT). U6 was used for normalization. (**b**–**d**) Expression of miR-18a-5p related to tumour size, sex and pathological stage. (**e**) The relative expression level of miR-18a-5p in lung cancer cell lines and BEAS-2B cells (control) was measured by qRT-PCR (*n*=3 independent experiments). Error bars represent the mean±S.E.M. *P<0.05, **P<0.01, ***P<0.001

In order to investigate the role of miR-18a-5p in lung carcinogenesis, its expression level was detected in 63 NSCLC patient cases. The data revealed that compared with corresponding non-tumour lung tissues, miR-18a-5p was upregulated (Figure 1a). In these patient tissues, elevated IRF2 expression levels was likely related to tumour size (*P*=0.052383, Figure 1b), but not associated with gender (Figure 1c) and pathological stage (Figure 1d). The expression of miR-18a-5p in NSCLC cell lines was also examined, BEAS-2B cell line was control. It was observably upregulated in A549, H1299, H23 and H1650 cells (Figure 1e). These results indicated that miR-18a-5p could promote NSCLC carcinogenesis.

### MiR-18a-5p promotes cell proliferation and migration of NSCLC

**Figure 2:**
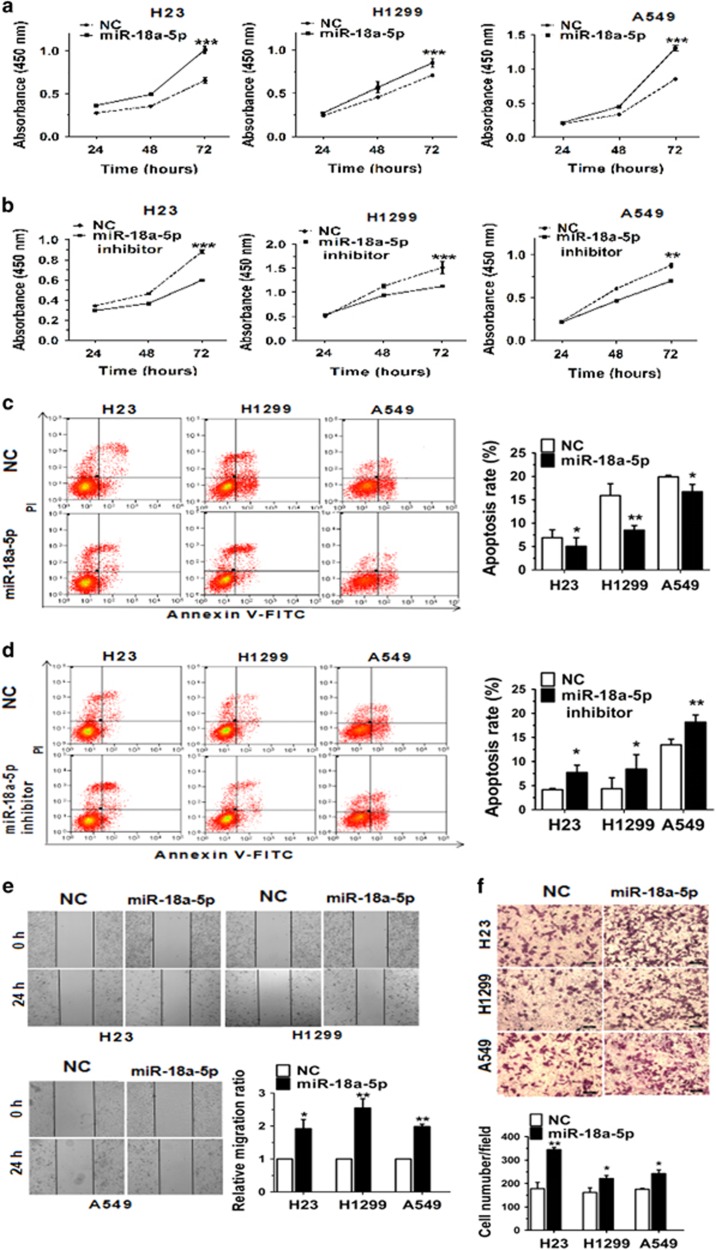
MiR-18a-5p can promote cell proliferation and migration of NSCLC cell lines. (**a**, **b**) H23, H1299 and A549 cells were transfected with NC or miR-18a-5p mimic/NC or miR-18a-5p inhibitor, and cell proliferation was determined by CCK-8. (**c**, **d**) The apoptosis distributions of H23, H1299 and A549 cells were transfected with NC or miR-18a-5p mimic/NC or miR-18a-5p inhibitor for 48 h then detected by flow cytometry. (**e**, **f**) 48 h after transfected with NC or miR-18a-5p mimic/NC or miR-18a-5p inhibitor, the results of wound healing assays and Transwell assays in H23, H1299 and A549 cells. *n*=3–4 independent experiments, Error bars represent the mean±S.E.M. *P<0.05, **P<0.01, ***P<0.001

To study the effect of miR-18a-5p on NSCLC cell proliferation, H23, H1299 and A549 cells were transfected with miR-18a-5p mimic/inhibitor and subjected to quantitative real-time PCR (qRT-PCR) analysis, the results from which validated that transfection of miR-18a-5p mimic/inhibitor did increase/decrease its level in three cell lines (Supplementary Figure S1). Proliferation of NSCLC cells was assessed by the Cell Counting Kit-8 (CCK-8) (Figures 2a and b). Cellular proliferation data showed that transfection with miR-18a-5p mimic exhibited a significant increase to cell growth after 72 h, while miR-18a-5p inhibitor decreased cell proliferation remarkably in the same culture conditions. Furthermore, the Flow Cytometry assessed that the apoptosis rate in three cell lines transfected with miR-18a-5p mimic/inhibitor was decreased/increased visibly (Figures 2c and d). Additionally, 48 h after transfection with miR-18a-5p mimic, the results of wound healing and Transwell showed that the migration ability of cells was augmented distinctly (Figures 2e and f). These results demonstrated that miR-18a-5p could promote the proliferation and migration ability, inhibit the apoptosis of NSCLC cells.

### MiR-18a-5p directly targets IRF2

**Figure 3:**
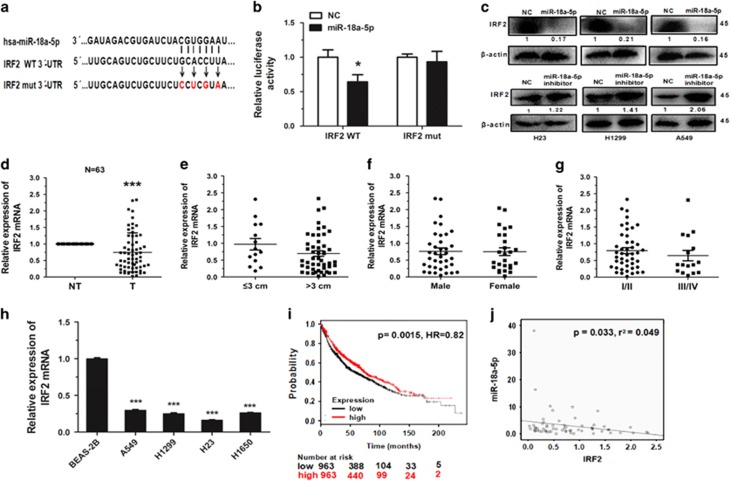
IRF2 is a direct target of miR-18a-5p. (**a**) IRF2 WT 3'-UTR contains predicted miR-18a-5p binding sites. The figure shows alignment of miR-18a-5p with the IRF2 WT 3'-UTR, with arrows indicating the mutagenesis nucleotides. (**b**) Dual luciferase reporter assay. HEK293T cells were co-transfected with luciferase reporter constructs containing the pGL3-IRF2 WT 3'-UTR (IRF2 WT) and the pGL3-IRF2 mut 3'-UTR (IRF2 mut) with miR-18a-5p mimic or NC mimic. Relative firefly luciferase expression is displayed, normalized to Renilla luciferase expression, *n*=3 independent experiments. (**c**) Immunoblotting analysis of IRF2 levels in H23, H1299 and A549 cells transfected with NC mimic/miR-18a-5p mimic or NC inhibitor/miR-18a-5p inhibitor for 48 h. (**d**) The relative expression of IRF2 mRNA from qRT-PCR of corresponding non-tumour tissues (NT) and tumour tissues (T). 18 S was used for normalization. (**e-g**) Expression of IRF2 related to tumour size, sex and pathological stage. (**h**) The relative expression of IRF2 mRNA in lung cancer cell lines or a pulmonary epithelial cell line, *n*=3 independent experiments. (**i**) The effect of IRF2 expression levels on the overall survival in 1926 lung cancer patients (Kaplan–Meier Plotter online database) was analysed and Kaplan–Meier plots were generated using a Kaplan–Meier plotter (http://www.kmplot.com). (**j**) MiR-18a-5p had a negative correlation with IRF2 according to Pearson correlation coefficient. Error bars represent the mean±S.E.M. * *P*<0.05, **P<0.01, ***P<0.001

Using the TargetScan prediction programs (www.targetscan.org), IRF2 was selected as a potential target of miR-18a-5p. To verify whether IRF2 is a direct target of miR-18a-5p or not, the IRF2 wild type 3'-UTR (IRF2 WT 3'-UTR) was cloned into the pGL3 vector (pGL3-IRF2 WT 3'-UTR), downstream of the luciferase open reading frame. In order to validate target specificity, we mutated the miR-18a-5p binding sites (Figure 3a) and conducted site-directed mutagenesis for IRF2 WT 3'-UTR using the QuikChange Mutagenesis kit. The results showed a significant, more than 30% decrease to the relative luciferase activity of the reporter gene in HEK293T cells co-transfected with pGL3-IRF2 WT 3'-UTR and miR-18a-5p mimic. Conversely, co-transfection of miR-18a-5p with pGL3-IRF2 mut 3'-UTR resulted in imperceptible change in luciferase activity, suggesting miRNA target 3'-UTR specificity (Figure 3b). Furthermore, we observed that miR-18a-5p reduced IRF2 expression in cells (Figure 3c).

To determine whether the expression of IRF2 was associated with miR-18a-5p in NSCLC or not, the expression of IRF2 was investigated in the same pairs of tissues. The results indicated that IRF2 expression was downregulated in tumour tissues (Figure 3d), and likely related to tumour size (*P*=0.080892, Figure 3e), but negative correlation with sex and pathological stage (Figures 3f and g). Then the expression levels of IRF2 mRNA was measured in several NSCLC cell lines, and found that expression of IRF2 was much lower in NSCLC cells than BEAS-2B control cells (Figure 3h). Therefore, according to the results of analysis using the Pearson correlation coefficient, we surmised that miR-18a-5p is negatively correlated with IRF2 (Figure 3j).

In order to analyse the effect of IRF2 expression of lung cancer patients, we used the Kaplan–Meier plotter online database (www.kmplot.com/analysis) to generate a survival curve of NSCLC patients with low or high expression of IRF2 (Figure 3i). Of the 1926 cases, we found that NSCLC patients with low expression of IRF2 had lower survival rates.

### IRF2 functions to suppress NSCLC

**Figure 4:**
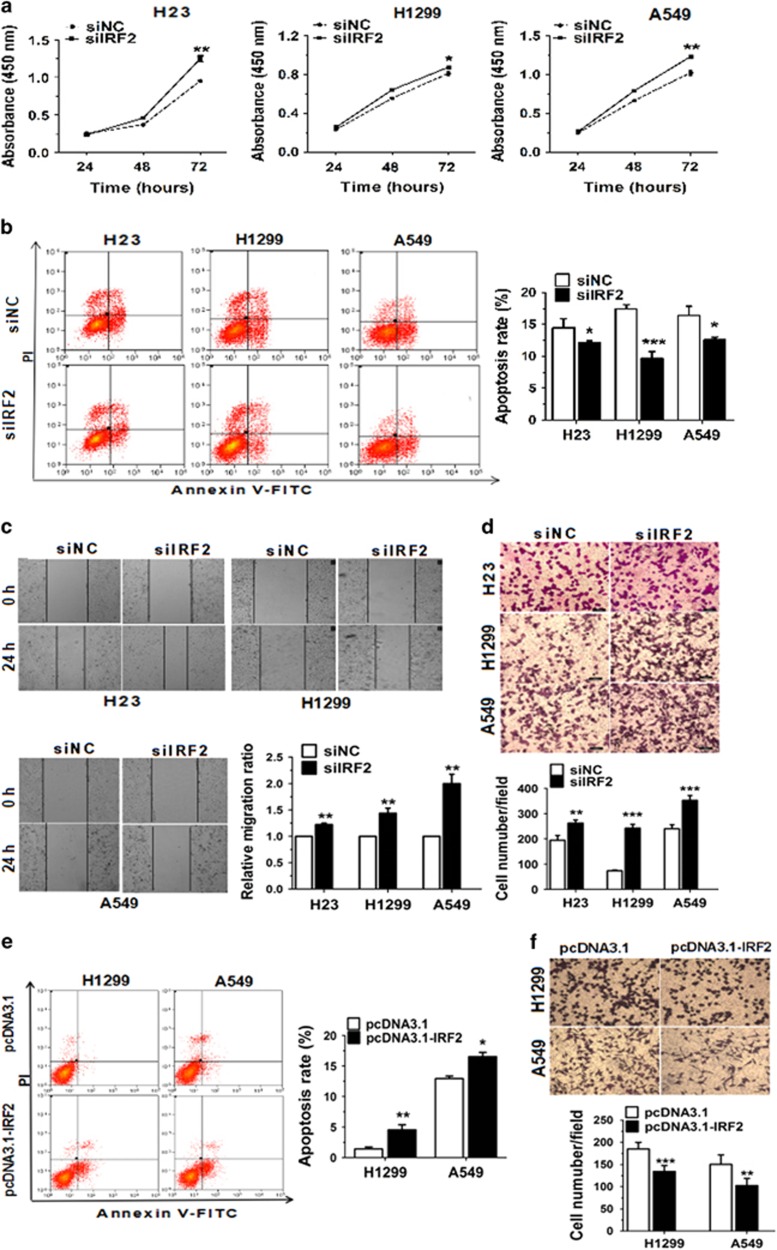
IRF2 functions to suppress NSCLC. (**a**) H23, H1299 and A549 cells were transfected with siNC or siIRF2, and cell proliferation was determined by CCK-8. (**b**) The apoptosis distributions of H23, H1299 and A549 cells were transfected with siNC or siIRF2 for 48 h then detected by flow cytometry. (**c, d**) 48 h after transfected with siNC or siIRF2, the results of wound healing assays and Transwell assays in H23, H1299 and A549 cells. (**e, f**). H1299 and A549 cells were co-transfected with miR-18a-5p mimic and pcDNA3.1/pcDNA3.1-IRF2 vector then cell migration ability subject to Transwell migration and invasion assays, and cell apoptosis was detected 48 h later by flow cytometry analyses. *n*=3–4 independent experiments, error bars represent the mean±S.E.M. *P<0.05, **P<0.01, ***P<0.001

To determine whether the biological effects of IRF2 and miR-18a-5p were same or not, IRF2 was knocked down using siRNA and then the cell proliferation, apoptosis and migration ability were detected. After 48 h, the expression of IRF2 mRNA and protein decreased by 50% in cells transfected with siIRF2 (Supplementary Figures S2A and S2B). Moreover, the results of CCK-8 assays showed that the proliferation capacity of NSCLC cells was significantly augmented after treatment with siIRF2 (Figure 4a). Correspondingly, the apoptosis rate was inhibited (Figure 4b). The results also showed that the ability of cell migration was improved 48 h after knockdown of IRF2 (Figures 4c and d).

To investigate whether the effects of miR-18a-5p was mediated through IRF2, we transfected negative control (pcDNA3.1) or IRF2 expression vector (pcDNA3.1-IRF2) into A549 or H1299 cells, which overexpressed miR-18a-5p. The protein expression of IRF2 was increased following transfected in A549 or H1299 cells (Supplementary Figure S2C). We also analysed the effects of IRF2 overexpressing on cell apoptosis and migration ability. Dramatically, the results indicated that IRF2 reversed the function of miR-18a-5p overexpression, which suppressed apoptosis and promoted migration ability in A549 and H1299 cells (Figures 4e and f). In conclusion, these data suggested that the effects of IRF2 suppresses NSCLC by promoting cell apoptosis, inhibiting cell proliferation and migration ability.

### MiR-18a-5p promotes autophagy in NSCLC

**Figure 5:**
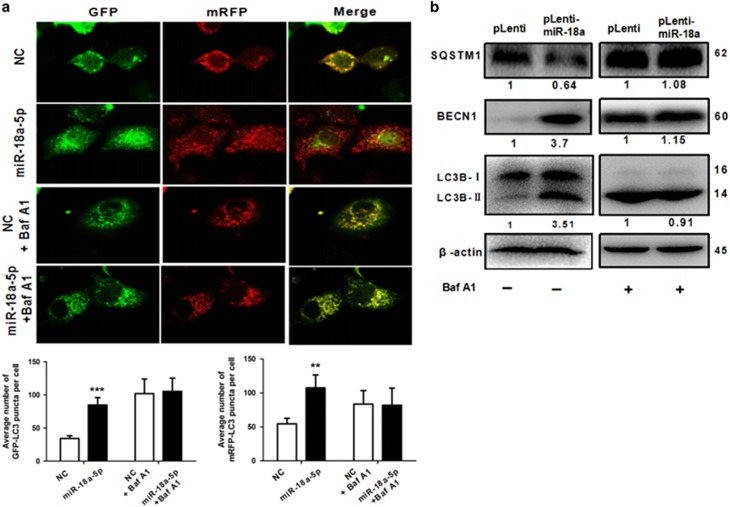
MiR-18a-5p promotes autophagy in NSCLC. (**a**) MiR-18a-5p boosted GFP/mRFP-LC3 dot accumulation in A549 cells. But, Baf A1 inhibited the function of MiR-18a-5p. Cells were transfected with NC or miR-18a-5p mimic and treated for 100 nM Baf A1 for 24 h (**b**) Immunoblotting analysis of protein levels of the miR-18a-stably-overexpressing A549 cells treated for 100 nM Baf A1 for 24 h. *n*=3 independent experiments, error bars represent the mean±S.E.M.*P<0.05, **P<0.01, ***P<0.001

**Figure 6:**
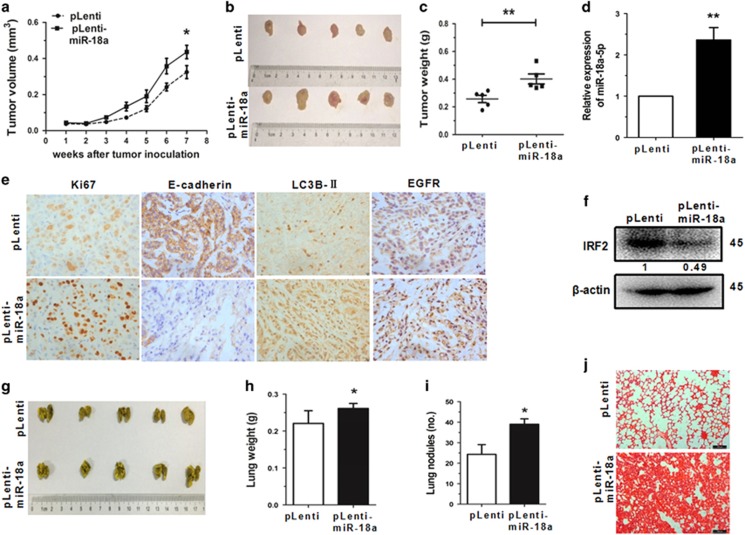
MiR-18a-5p increases tumour growth and metastasis *in vivo*. (**a-c**) MiR-18a-stably-overexpressing A549 cells (pLenti-miR-18a) and control cells (pLenti) were injected into nude mice. The tumour growth curve is shown in Figure 6a. The xenograft tumours on the nude mice are shown in Figure 6b, and the tumour weight shown in Figure 6c. (**d**) The expression of miR-18a-5p was detected by qRT-PCR in miR-18a stably overexpressing A549 cells and negative control cells. (**e**) The expression of Ki67, E-cadherin, LC3B-II and EGFR in tumour tissues were measured by immunohistochemistry. (**e**) The IRF2 expression in tumour tissues was assessed by western blot. (**g-i**) The lungs of mice with metastasis nodes are displayed in Figure 6g. The lung weight and metastasis nudes are displayed in Figures 6h and i. (**j**) Histopathology of metastases induced by mouse lung tissues with haematoxylin and eosin staining. Error bars represent the mean±S.E.M. *P<0.05, **P<0.01, ***P<0.001

Autophagy has a two-sided effect in tumours.^14^ That is the reason why we investigated the effect of miR-18a on autophagy. As shown in Figure 5a, overexpression of miR-18a-5p significantly induced GFP/mRFP-LC3 dot accumulation. Notably, lipid conjugation of free LC3-I to the autophagic membrane-associated LC3-II was boosted and enhanced BECN1 in the extracts of cells following miR-18a overexpression (Figure 5b). Conversely, the expression levels of SQSTM1 was decreased (Figure 5b). We also detected the autophagy *in vivo* by immunohistochemistry which indicated that LC-3B-II in the xenograft tumour tissues was elevated (Figure 6e). To determine whether miR-18a-5p regulates autophagosome formation or autophagy flux, Bafilomycin A1 (Baf A1), which is a known inhibitor of the latter stages of autophagy, has been used in the experiments. Interestingly, the effect of miR-18a-5p on autophagy is less obvious (Figures 5a and b). Consequently, such findings demonstrated that miR-18a enhanced not only autophagosome formation but autophagy flux.

### MiR-18a-5p increases tumour growth and metastasis *in vivo*

Based on the role of miR-18a-5p in NSCLC cells, its functions were also examined *in vivo*. Nude mice were injected subcutaneously with A549 cells (5 × 10^6^) stably overexpressing miR-18a (pLenti-miR-18a) or control (pLenti) 7 weeks later the subsequent tumours were assayed. The results showed that A549 xenografts significantly increased in tumour growth. The miR-18a-5p overexpressing cells also promoted tumour size and weight after 7 weeks post-implantation (Figures 6a and c). Furthermore, a conspicuous upregulation in the expression of miR-18a-5p (Figure 6d) and decreased IRF2 expression level (Figure 6f) was observed in tumour tissues from the pLenti-miR-18a group. The expression of Ki67, E-cadherin and epidermal growth factor receptor (EGFR) in the xenograft tumour tissues were measured using immunohistochemistry. Results showed a significant upregulation of Ki67 and EGFR in the tumour tissues stably overexpressing miR-18a, accompanied by reduction of E-cadherin expression (Figure 6e).

To investigate how miR-18a effected tumour metastasis *in vivo*, cells were injected into the tail vein of nude mice. After 7 weeks of tail vein injection, we observed that the overexpression groups generating massive and confluent metastatic nodes, the control group generated fine and scattered metastatic nodes (Figures 6f–h). The haematoxylin and eosin staining showed that the tumour nests derived from overexpression groups exhibited a large area of lung tissue destruction and/or necrosis, whereas control group formed fewer and smaller tumour nests (Figure 6i). These data indicated that miR-18a-5p could promote tumour growth, metastasis and autophagy.

## Discussion

MicroRNAs play important roles in cell proliferation and carcinogenesis. Some microRNAs function as oncogenes, while others as suppressor genes. Our lab confirmed that miR-34a,^15^ miR-32^16^ and miR-486-5p^17^ could be tumour suppressor genes, while miR-150^18^ could be an oncogene. They are involved in much more complex regulation network in tumorigenesis of lung cancer.

In this study, we confirmed that miR-18a-5p promotes cell proliferation and decreases migration by directly targeting IRF2. Because dysregulation of the IRF signalling pathway is one of the most common changes found in human cancers and diseases,^19^^20^ including NSCLC. IRF2 is considered as one of the most desirable targets for cancer therapies.^21, 22, 23^

Recently, autophagy has been generally reported to play a key role in different human diseases, most notably in cancer.^24, 25^ As a very important physiological process of cancer cells, autophagy is involved in cell cycle, cell growth, cell invasion and metastasis.^26, 27, 28, 29^ We found that miR-18a-5p as an oncogene-induced autophagy in NSCLC. The more validated the autophagy in association with cancer may unveil novel strategies for tumour therapy. The EGFR is an important factor involved in the progression of NSCLC.^30, 31^ EGFR affects numerous systems involved in oncogenesis.^32^ It has been proposed as an attractive and promising target for anti-cancer treatment.^33^ In our cases, overexpression of miR-18a was upregulation of autophagy and positive correlation with expression of EGFR *in vivo*.

In summary, our study focused on the mechanism of miRNA-18a-5p and its potential as a diagnostic biomarker in NSCLC.^34^ Our data supported the hypothesis that miR-18a-5p could promote carcinogenesis that anti-miRNA could be potential new treatment for NSCLC. A separate study reported that miR-18a-5p increases cell growth of tumours,^35, 36, 37^ which substantiated the concept that miR-18a-5p might be less tumour-specific than other miRNAs, making it a more dependable therapeutic strategy.^38^

**Figure 7:**
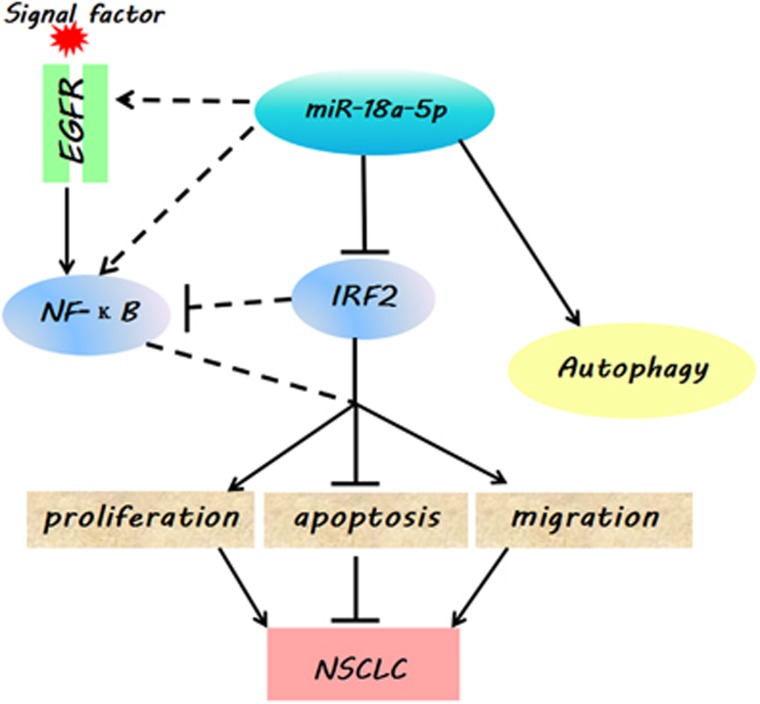
A model depicting the effect of miR-18a-5p in NSCLC. The miR-18a-5p prompts oncogenic function in NSCLC through downregulation of IRF2. Overexpression of miR-18a-5p significantly induced autophagy and positive correlation with levels of EGFR

Overall, based on these results, we concluded that miR-18a-5p could drive cancer by directly targeting IRF2 (Figure 7), and might also have a close correlation between the p53 and NF-*κ*B signalling pathway (Supplementary Figure S3).^39, 40, 41^ Our findings provided a potential understanding of the mechanism how miRNAs affect the oncogenesis of lung cancer, which could be employed as drug targets for diagnostics and therapeutic treatments in the future.

## Materials and methods

### Cell culture

The human lung epithelial cell line BEAS-2B and the human NSCLC cell lines A549 were obtained from the Cell Bank, China Academy of Sciences (Shanghai, China). The H23, H1299 and H1650 cell lines were obtained from the American Type Culture Collection (ATCC, Manassas, VA, USA). A549 cells were all cultured in DMEM medium (Corning Cellgro, Manassas, VA, USA) supplemented with 10% fetal bovine serum (FBS, Gibco, Gaithersburg, MD, USA). H23, H1299 and H1650 cells were cultured in RPMI 1640 medium (Corning Cellgro) supplemented with 10% FBS (Gibco). BEAS-2B cells were cultured in LHC medium (Gibco) supplemented with 10% FBS (Gibco). Culture conditions for all cells were at 37 °C in a humidified 5% CO_2_ atmosphere.

### Tissue samples

Sixty-three pairs of human NSCLC samples were obtained from the Shanghai Chest Hospital affiliated with Shanghai Jiao Tong University, and with the approval from the ethics committee of Shanghai Chest Hospital. Details of all samples used in this paper are listed in Supplementary Table S1.

### Transfection

H23, H1299 and A549 cells were transiently transfected with miR-18a-5p mimic, negative control mimic (NC), miR-18a-5p inhibitor, IRF2 siRNA (siIRF2), or negative control siRNA (siNC) (RIBOBIO, Guangzhou, China) using Invitrogen™ Lipofectamine 2000 (Life Technologies, New York, USA), according to the manufacturer’s recommendations. After 24–48 h post-transfections, cells were used for subsequent experiments, including assays for proliferation and cell cycle analysis.

### RNA isolation, reverse transcription and quantitative real-time PCR

Total RNA was isolated using Trizol Reagent (Sangon Biotech, Shanghai, China) and cDNA synthesis was performed with the SYBR PrimeScript miRNA RT-PCR Kit and PrimeScript RT Master Mix (Takara Biotech, Otsu, Japan), following the manufacturer’s instructions for each reagent or kit. Analysis of miRNA and mRNA by qRT-PCR using SYBR GreenII (Takara Biotech) was performed according to manufacturer’s protocol, with a CFX96^TM^ Real-time System (Bio-Rad, California, USA). Relative quantification of miR-18a-5p was obtained by normalization to U6 expression levels, and relative quantification of IRF2 was obtained by normalization to 18 S rRNA expression levels. The expression levels of mRNAs and miRNAs were determined by the 2^-ΔΔCt^ method for relative quantification of gene expression. ΔCt and ΔΔCt were calculated using the following formulae: ΔCt=Ct_miR-18a-5p_ – Ct_U6_ or Ct_IRF2_ – Ct_18S_ and ΔΔCt=ΔCt_case_ – ΔCt_control_.

### Construction of recombinant expression vectors

In brief, pri-miR-18a sequence was digested with BamH I and Xhol I, then cloned into the pLenti vector (Invitrogen, Carlsbad, CA, USA) and named pLenti-miR-18a. The CDS of IRF2 sequence were cloned into the pcDNA3.1(−) plasmid, formed pcDNA3.1-IRF2. The primer sequences used in this study are shown in Supplemantary Table 2.

### Cell proliferation analysis

Cell proliferation was measured with the CCK-8 assay kit (Dojindo, Tokyo, Japan). Six hours after transfection, cells were plated into a 96-well microplate (Corning Incorporated, New York, USA) and incubated at 37 °C in 5% CO_2_. Each data point represents the measurement of three replicate wells. After 24, 48 and 72 h of culture, 8 μl of CCK-8 solution was added to each well with 100 μl of serum-free medium, then incubated for 90 min. Finally, the absorbance was measured at 450 nm using a multi-function enzyme-linked analyser, FLx8 (BioTek, Vermont, USA).

### Cell apoptosis assay

After 48 h of transfection, H23, H1299 and A549 cells was harvested for assay. An Annexin V-fluorescein isothiocyanate (FITC) apoptosis detection kit (BD Pharmingen, San Diego, CA, USA) was used according to the manufacturer’s instruction in order to determine the level of cell apoptosis. Apoptotic cells were analysed using a MoFlo XDP flow cytometer (Beckman Coulter, Inc., Brea, CA, USA).

### Cell migration assay

Migration of H23, H1299 and A549 cells *in vitro* were assayed using a Transwell chamber (Corning Incorporated, New York, USA) with a polycarbonic membrane (8 μm pore size). After 24 h of transfection, 1 × 105 cells added to the upper chamber with 100 μl of serum-free medium, and 600 μl of medium with 10% FBS was added to the lower chamber. The cells were cultured for 24 h at 37 °C. Then, chambers washed twice by PBS. Last, count the cell number after staining with crystal violet.

For wound healing assay, the transfected cells were seeded into 24 wells and cultured to 100%. Then, a single-scratch wound was made in the centre of the well by sterile pipette tip. Cell debris was removed by washing with PBS, and cells were allowed to grow in the serum-free medium. The cells migration distance was assessed and photographed at 24 h by microscope (Nikon,Tokyo, Japan).

### Dual luciferase reporter assay

The IRF2 WT 3'-UTR firefly luciferase construct (pGL3-IRF2 WT 3'-UTR) was generated by inserting a fragment from 1502 bp to 1775 bp of human IRF2 3'-UTR into the *Xba*I/*Eco*RI sites of the pGL3 luciferase report vector. The pGL3-IRF2 mut 3'-UTR construct was generated by mutation of the complementary seed sequence of the miR-18a-5p binding region. HEK293T cells were co-transfected with 150 ng pGL3-IRF2 WT 3'-UTR or pGL3-IRF2 mut 3'-UTR luciferase reporter, and 15 ng Renilla luciferase reporter (pRL), and a final concentration of 100 nM of miRNA NC or miR-18a-5p mimic using Invitrogen™ Lipofectamine 2000 (Life Technologies). Cells were incubated for 48 h and luciferase activity was assayed with an Orion II Microplate Illuminometer (Titertek-Berthold, South San Francisco, CA, USA) according to the manufacturer’s instructions. Firefly luciferase units were normalized against Renilla luciferase units to control for transfection efficiency. Relative activities were expressed as the fold-change in luciferase activity.

### Western blot analysis

Total protein was extracted using RIPA lysis buffer (CWBIO, Beijing, China) and quantified by Bradford assay.^42^ Equal amounts of protein from each sample were subjected to SDS-PAGE electrophoresis and transferred to a polyvinylidene fluoride membrane (Millipore Corporation, Billerica, MA, USA). The membrane was then soaked in tris-buffered saline with Tween-20 (TBST, 150 mM NaCl, 20 mM Tris-HCl pH 8.0, 0.05% Tween-20) containing 5% bovine serum albumin for 1 h at room temperature, followed by gentle shaking and subsequent incubation with specific antibody against IRF2 or *β*-actin (1:1000, Cell Signaling Technology, Danvers, MA, USA) at 4 °C overnight. Afterwards, the membrane was washed and incubated with a horseradish peroxidase (HRP)-conjugated secondary antibody (1:10000, Santa Cruz Biotechnology, Santa Cruz, CA, USA) for 1 h at room temperature. Protein bands were detected using a chemiluminescent HRP substrate (Millipore Corporation) and analysed by Image Lab analysis software (Bio-Rad). IRF2 was normalized to *β*-actin and expressed as a percentage of the control.

### Lentivirus construction and infection

pLenti-miR-18a or pLenti vector was co-transfected into HEK293T with psPAX2 and pMD2G. Viral particles were collected 48 h and 72 h later, centrifuged them together at 4000 rpm for 5 min at 4º, then filtered through 0.45 μm filters. The cells were transfected with pLenti-miR-18a or pLenti and sorted for green fluorescence via flow cytometry.

### Autophagy analyses by confocal microscopy

Forty-eight hours after transfection of miR-18a-5p mimic and pGTLV-EGFP-mRFP-LC3 virus, counted the GFP-LC3 dot total number of transfected cells. The cells observed under the Laser Scanning Confocal Microscopy (Occult International Ltd, Germany) counted GFP-LC3 dot positive cells number.

### Tumour xenograft assay/*in vivo* metastatic assay

Six-week-old female nude mice were purchased from the SLRC Laboratory Animal Center (Shanghai, China). The animals were housed randomly to two groups, and each nude mice was injected subcutaneously into the right flank with 1 million A549 cells stably overexpressing miR-18a (pLenti-miR-18a) or control (pLenti) in 100 μl DMEM medium for establishing the subcutaneous tumour xenograft model. Tumour were measured weekly by calipers, and tumour volumes were calculated as formula: volume=length × width^2^/2. One million cells in 100 μl DMEM medium were injected into the tail vein, and 7 weeks later, the mice were killed and lung tissues were isolated. Evaluation of lung tissue weights was used to quantify metastasis. Lung tissues were subjected to serial sectioning and then haematoxylin and eosin staining. Pathological changes were observed under a light microscope (Nikon, Tokyo, Japan). All experimental protocols were approved by the Institutional Animal Care and Use Committee of Shanghai University (Shanghai, China). Animals used in this study were humanely treated.

### Immunohistochemistry

Tumour growth was assessed by immunohistochemical staining of Ki-67, E-cadherin and LC3-II. Tumour biopsies were fixed with formalin, embedded in paraffin and cut into sections of about 4 μm. Samples were then deparaffinized and dehydrated with xylene and graded alcohols, and subsequently rehydrated with demineralized water. Immunohistochemistry was performed using microwave pre-treatment of slides for antigen retrieval. Primary antibodies against Ki-67, E-cadherin and LC3-II (1:500, Cell Signaling Technology) were applied, together with goat anti-rabbit HRP-conjugated antibodies, and proteins were visualized *in situ* with a 3,3′-diaminobenzidine reaction solution.

### Statistical analysis

Statistical analyses were performed using SPSS v.19.0 software and graph presentations were completed using GraphPad Prism 5 Software. Results are represented as the mean±SEM, and the difference between two experimental groups was evaluated using Student’s *t*-test, with statistical significance defined as *P*<0.05.
