# Supplementary material for: MicroRNA-18a-5p functions as an oncogene by directly targeting IRF2 in lung cancer
Source: Cell Death Dis. 2017 May 4;8(5):e2764–. doi: 10.1038/cddis.2017.145 (PMC5520692; doi:10.1038/cddis.2017.145)
Supplement: Supplementary Figure Legends [file cddis2017145x1.doc]

**Supplementary Figure S1. The expression of miR-18a-5p. Referred to Figure 2**

**A and B.**48 h after transfection of negative control (NC) or miR-18a-5p mimic, NC or miR-18a-5p inhibitor, the relative expression levels of miR-18a-5p in H23, H1299 and A549 cells was measured by qRT-PCR.

n=3 independent experiments, Error bars represent the mean±S.E.M. * P<0.05, ** P<0.01, *** P<0.001

**Supplementary Figure S2. The expression of IRF2. Referred to Figure 4**

**A.** 48 h after transfection of siNC or siIRF2, the relative expression levels of miR-18a-5p in H23, H1299 and A549 cells was measured by qRT-PCR.

**B.** The variation of IRF2 in H23, H1299 and A549 cells transfected with siNC or siIRF2 incubated at 37 °C for 48 h were measured by Western blot.

**C.** Immunoblotting analysis of IRF2 levels in pcDNA3.1 or pcDNA3.1-IRF2 vector transfected H1299 and A549 cells incubated at 37 °C for 48 h.

n=3-4 independent experiments, Error bars represent the mean±S.E.M. * P<0.05, ** P<0.01, *** P<0.001

**Supplementary Figure S3. The expression of P65 after transfected with siNC or siIRF2 in NSCLC cells. Referred to Figure 7**

**A.** The variation of P65 in H23, H1299 and A549 cells transfected with siNC or siIRF2 incubated at 37 °C for 48 h were measured by Western blot.
