# Supplementary figures and images for: MicroRNA-18a-5p functions as an oncogene by directly targeting IRF2 in lung cancer
Source: Cell Death Dis. 2017 May 4;8(5):e2764–. doi: 10.1038/cddis.2017.145 (PMC5520692; doi:10.1038/cddis.2017.145)

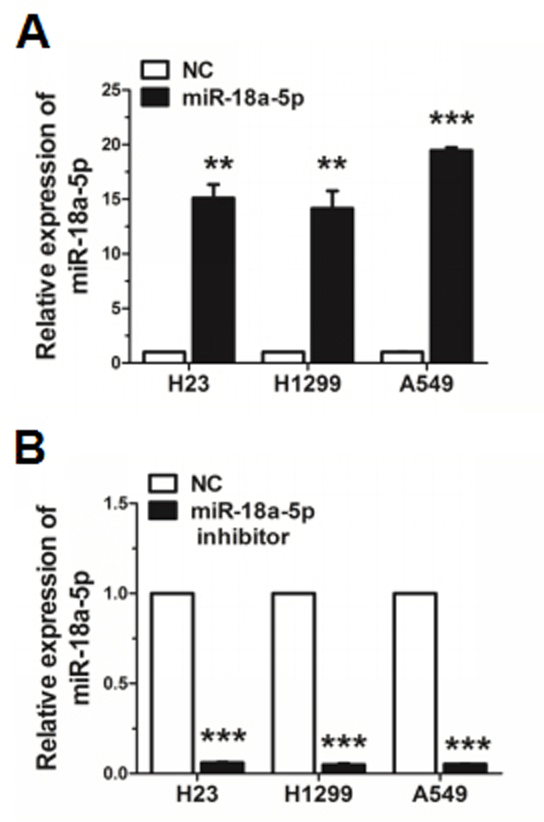

Supplement: Supplementary Figure 1 [file cddis2017145x2.tif]

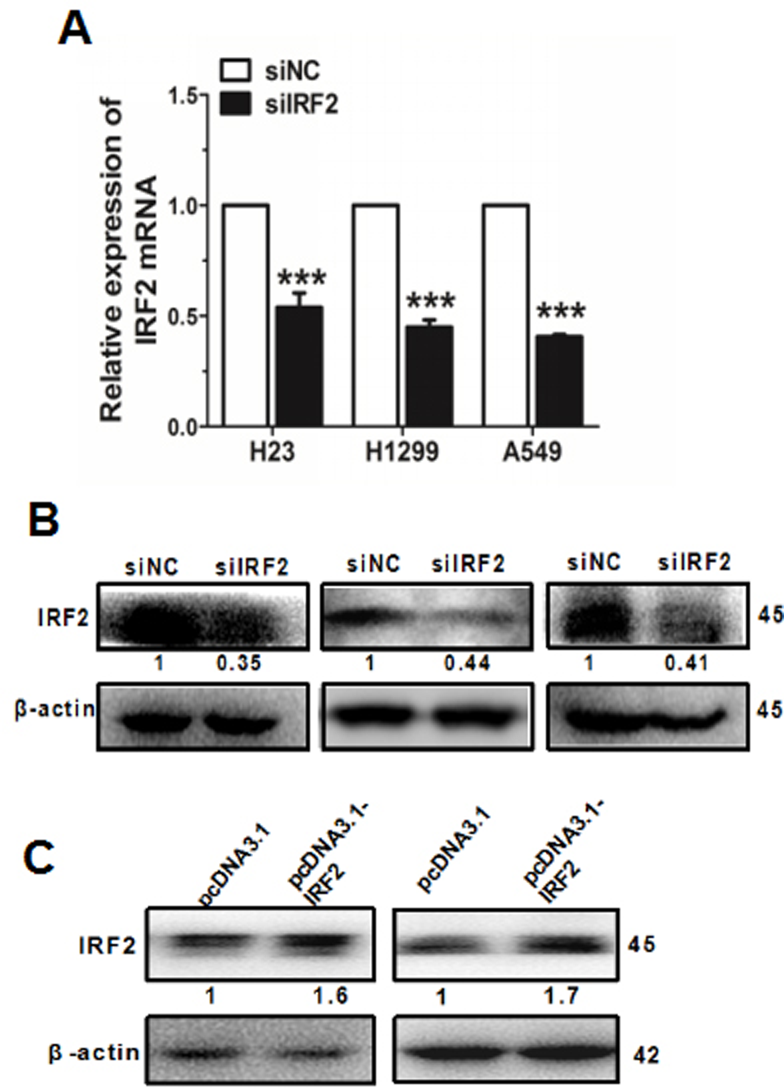

Supplement: Supplementary Figure 2 [file cddis2017145x3.tif]

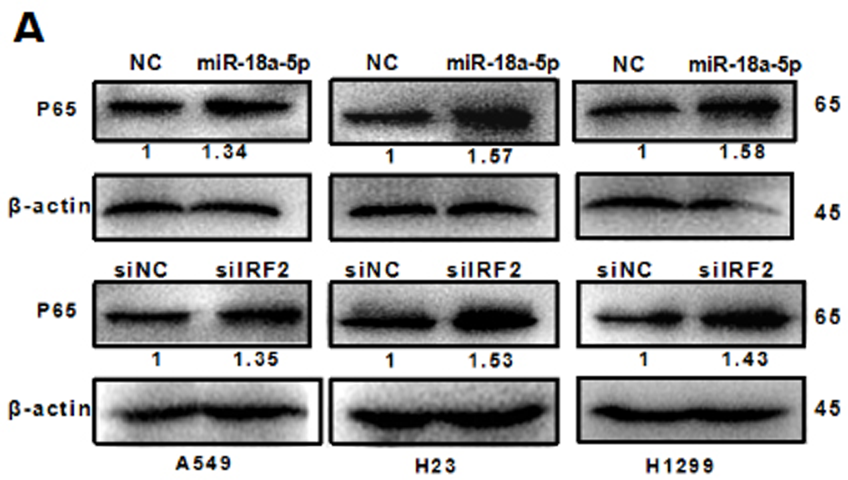

Supplement: Supplementary Figure 3 [file cddis2017145x4.tif]
