# Supplementary material for: MicroRNA-18a-5p functions as an oncogene by directly targeting IRF2 in lung cancer
Source: Cell Death Dis. 2017 May 4;8(5):e2764–. doi: 10.1038/cddis.2017.145 (PMC5520692; doi:10.1038/cddis.2017.145)
Supplement: Supplementary Table 1 [file cddis2017145x5.doc]

Supplementary table 1 The clinical-pathological features of 63 cases NSCLC patients

| NO. | Gender | Age | Histologic Type | Lymphatic Invasion | | pTNM |
| --- | --- | --- | --- | --- | --- | --- |
| 1 | M | 47 | Adenocarcinoma | Absent | T1bN0M0 | |
| 2 | M | 59 | Adenocarcinoma | Absent | T2aN0Ma | |
| 3 | F | 67 | Adenocarcinoma | Present | T2N1M0 | |
| 4 | M | 54 | Adenocarcinoma | Absent | T4N0M0 | |
| 5 | M | 70 | Adenocarcinoma | Absent | T3N0M0 | |
| 6 | M | 67 | Adenocarcinoma | Absent | T2aN0M0 | |
| 7 | F | 62 | Adenocarcinoma | Absent | T1aN0M0 | |
| 8 | F | 75 | Adenocarcinoma | Absent | T2aN0M0 | |
| 9 | F | 58 | Adenocarcinoma | Present | T2aN1M0 | |
| 10 | F | 65 | Adenocarcinoma | Present | T2aN0M0 | |
| 11 | M | 72 | Adenocarcinoma | Absent | T2aN0M0 | |
| 12 | M | 64 | Adenocarcinoma | Absent | T1aN0M0 | |
| 13 | F | 62 | Adenocarcinoma | Absent | T2aN0M0 | |
| 14 | M | 65 | Adenocarcinoma | Present | T4N2M0 | |
| 15 | F | 55 | Adenocarcinoma | Present | T2aN1M0 | |
| 16 | M | 50 | Adenocarcinoma | Absent | T2aN0M0 | |
| 17 | F | 60 | Adenocarcinoma | Absent | T1bN0M0 | |
| 18 | M | 67 | Adenocarcinoma | Absent | T2aN0M0 | |
| 19 | M | 71 | Adenocarcinoma | Absent | T1bN0M0 | |
| 20 | F | 64 | Adenocarcinoma | Absent | T2aN0M0 | |
| 21 | M | 62 | Adenocarcinoma | Absent | T2aN0M0 | |
| 22 | M | 58 | Adenocarcinoma | Absent | T2aN0M0 | |
| 23 | M | 59 | Adenocarcinoma | Absent | T2aN0M1a | |
| 24 | F | 72 | Adenocarcinoma | Absent | T2aN0M0 | |
| 25 | M | 67 | Adenocarcinoma | Absent | T2aN0M0 | |
| 26 | F | 64 | Adenocarcinoma | Present | T2aN2M0 | |
| 27 | M | 60 | Adenocarcinoma | Absent | T1bN0M0 | |
| 28 | F | 57 | Adenocarcinoma | Absent | T1bN0M0 | |
| 29 | F | 55 | Adenocarcinoma | Absent | T1bN0M0 | |
| 30 | F | 53 | Squamous cell | Absent | T3N0M0 | |
|  |  |  | carcinoma |  |  | |
| 31 | M | 54 | Squamous cell | Present | T4N2M0 | |
|  |  |  | carcinoma |  |  | |
| 32 | M | 58 | Squamous cell | Present | T3N2M0 | |
|  |  |  | carcinoma |  |  | |
| 33 | M | 65 | Squamous cell | Absent | T1bN0M0 | |
|  |  |  | carcinoma |  |  | |
| 34 | M | 61 | Squamous cell | Absent | T1bN0M0 | |
|  |  |  | carcinoma |  |  | |
| 35 | M | 67 | Squamous cell | Present | T2aN1M0 | |
|  |  |  | carcinoma |  |  | |

Table 1 The clinical-pathological features of 63 cases NSCLC patients

| NO. | Gender | | Age | | Histologic Type | | Lymphatic Invasion | | pTNM | |  |
| --- | --- | --- | --- | --- | --- | --- | --- | --- | --- | --- | --- |
| 36 | M | | 67 | | Squamous cell | | Present | | T2aN1M0 | |  |
|  |  | |  | | carcinoma | |  | |  | |  |
| 37 | M | | 69 | | Squamous cell | | Absent | | T2aN0M0 | |  |
|  |  | |  | | carcinoma | |  | |  | |  |
| 38 | M | | 71 | | Squamous cell | | Present | | T2aN2M0 | |  |
|  |  | |  | | carcinoma | |  | |  | |  |
| 39 | M | | 61 | | Squamous cell | | Absent | | T1bN0M0 | |  |
|  |  | |  | | carcinoma | |  | |  | |  |
| 40 | M | | 72 | | Adenocarcinoma | | Absent | | T2aN0M0 | |  |
| 41 | M | | 51 | | Adenocarcinoma | | Absent | | T1bN0M0 | |  |
| 42 | M | | 51 | | Adenocarcinoma | | Absent | | T1aN0M0 | |  |
| 43 | F | | 55 | | Adenocarcinoma | | Absent | | T1bN0M0 | |  |
| 44 | M | | 65 | | Combined Small Cell | | Present | | T2bN2M0 | |  |
|  |  | |  | | carcinoma | |  | |  | |  |
| 45 | M | | 77 | | Squamous cell | | Present | | T2aN0M0 | |  |
|  |  | |  | | carcinoma | |  | |  | |  |
| 46 | M | | 71 | | Adenocarcinoma | | Present | | T1bN0M0 | |  |
| 47 | F | | 64 | | Adenocarcinoma | | Absent | | T2aN0M0 | |  |
| 48 | F | | 55 | | Adenocarcinoma | | Absent | | T1bN2M0 | |  |
| 49 | | M | | 62 | | Adenocarcinoma | | Absent | | T2aN0M0 | |
| 50 | | M | | 58 | | Adenocarcinoma | | Absent | | T2aN0M0 | |
| 51 | | F | | 77 | | Adenocarcinoma | | Present | | T4N3M0 | |
| 52 | | F | | 61 | | Adenocarcinoma | | Present | | T2aN1M0 | |
| 53 | | F | | 67 | | Adenocarcinoma | | Present | | T2aN1M0 | |
| 54 | | F | | 57 | | Adenocarcinoma | | Absent | | T4N0M0 | |
| 55 | | M | | 49 | | Adenocarcinoma | | Absent | | T1bN0M0 | |
| 56 | | M | | 65 | | Adenocarcinoma | | Absent | | T1bN0M0 | |
| 57 | | M | | 71 | | Squamous cell | | Absent | | T1bN0M0 | |
|  | |  | |  | | carcinoma | |  | |  | |
| 58 | | F | | 64 | | Adenocarcinoma | | Absent | | T3N0M0 | |
| 59 | | F | | 52 | | Adenocarcinoma | | Absent | | T3N0M0 | |
| 60 | | M | | 68 | | Squamous cell | | Absent | | T3N0M0 | |
|  | |  | |  | | carcinoma | |  | |  | |
| 61 | | F | | 57 | | Adenocarcinoma | | Present | | T3N3M0 | |
| 62 | | F | | 54 | | Adenocarcinoma | | Present | | T2aN3M0 | |
| 63 | | M | | 64 | | Adenocarcinoma | | Present | | T2bN2M0 | |
