# Supplementary material for: MicroRNA-18a-5p functions as an oncogene by directly targeting IRF2 in lung cancer
Source: Cell Death Dis. 2017 May 4;8(5):e2764–. doi: 10.1038/cddis.2017.145 (PMC5520692; doi:10.1038/cddis.2017.145)
Supplement: Supplementary Table 2 [file cddis2017145x6.doc]

**Supplementary Table S2: Primer sequences used in this study**

| **Primer Name** | **Primer Sequences(5**′ **- 3**′**)** |
| --- | --- |
| 18S RNA | Forward: AGGAATTCCCAGTAAGTGCG |
|  | Reverse: GCCTCACTAAACCATCCAA |
| U6 snRNA | Forward: CTCGCTTCGGCAGCACA |
|  | Reverse: AACGCTTCACGAATTTGCGT |
| miR-18a-5p(qPCR） | Forward:TAAGGTGCATCTAGTGCAGATAG |
| pri-miR-18a | Forward:CGGGATCCGTGAAGGCACTTGTAGCATT |
|  | Reverse:CCCTCGAGCAAAACTAACAGAGGACTGC |
| IRF2-mRNA(qPCR） | Forward: TCCATACAGGAAAGCATCA |
|  | Reverse: AGCATTCGGTAGACCCTGA |
| IRF2-3′-UTR | Forward: GCTCTAGACAACAAGGGCAGTGGAGGT |
|  | Reverse:CGGAATTCGGATGGGATGGGATAGGAA |
| IRF2-mut3′-UTR | Forward:GCTTCTCCTCGTAATCTTAAAGCACTTACAGAT  AGGCC |
|  | Reverse:AAGATTACGAGGAGAAGCAGACTGCAATGTCG  C |
| IRF2-CDS | Forward: CGGGATCCCATTTCCATTTCACATACCC |
|  | Reverse: GGGGTACC ACAAAACAAAGCCAAGAAG |
|  |  |
